# Transient unexpected improvement of AV conduction: What is the mechanism?

**Published:** 2006-07-01

**Authors:** Meir Friedman, Paul Schweitzer

**Affiliations:** Beth Israel Medical Center, New York, NY

This ECG was recorded from a 93 year old patient with a previously documented third degree AV block and an underlying LBBB. The twelve lead ECG demonstrates sinus bradycardia at a rate of 52 beats per minute, a PR interval  of 230 milliseconds, and a left bundle branch block (Figure 1).  In figure 2, a rhythm strip of leads V1, II and V5 shows sinus rhythm with AV block. Note that only P waves in the T wave of the paced beat are conducted.  The non-conducted P waves are followed by ventricular paced beats at an escape interval of 1200 milliseconds.

What is the mechanism of AV conduction in this patient?  The following possibilities should be considered:

1. Supernormal conduction
2. Wedensky phenomenon
3. Gap phenomenon
4. Peel back effect

In the sixties and seventies Pick et al [1], Schamroth [2] and others [3] suggested supernormal conduction as the most likely mechanism of unexpected  transient improvement of atrio-ventricular conduction defects.  This electrophysiological phenomenon was defined as better than expected conduction in patients with depressed conduction during a short interval in the ventricular cycle [1]. However, there is no definitive proof of supernormal conduction in humans.

The next possible explanation is the Wedensky effect.  This mechanism was first documented on the nerve-muscle preparation and was defined as a "stronger" stimulus, where in the case of AV block, a ventricular premature or paced beat, is followed by transient antegrade conduction by decreasing the refractoriness of the AV conduction [2]. Similarly to supernormal conduction, its existence in humans remains controversial.

According to Moe et al [4] and others [5], there are alternative explanations for electrocardiographic abnormalities suggestive of supernormal conduction or Wedensky phenomenon.  The first of such explanations is the Gap phenomenon. Experimental and clinical studies [4,5] showed that atrial premature beats with longer coupling intervals were blocked and earlier ones conducted. The explanation of Gap Phenomenon is conduction delay in the proximal part of the AV conduction system, causing recovery of its more distal portion. The gap phenomenon is an unlikely mechanism in our patient because the conducted P wave occurred after the paced beat rather than following a premature beat.

A more likely mechanism is the "peeling back" effect.  It is assumed that pre-excitation of the AV node by a ventricular or junctional beat shortens the absolute refractory period of the AV or the His-Purkinje system and allows conduction of a supraventricular impulse [4].

The final question is the site of AV block.  Because the patient has a left bundle branch block the possibility of infranodal block has to be considered.  However, without His bundle recording, the site of AV block remains uncertain.

## Figures and Tables

**Figure 1:**
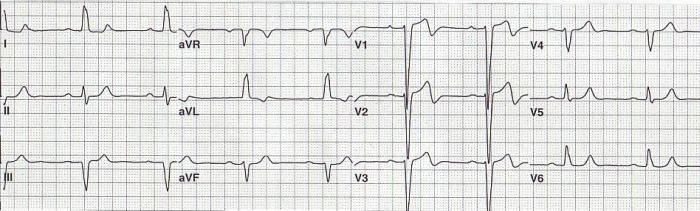
Shows sinus bradycardia at a rate of 52 beats per minute, PR interval of 230 milliseconds and a left bundle branch block.

**Figure 2:**
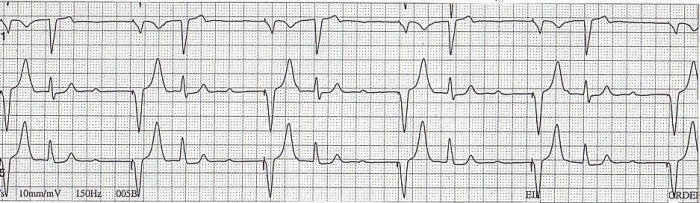
This is a rhythm strip where lead V1, II and V5 demonstrates 2:1 AV block. The P waves in the T wave of the paced beats are conducted while the others are not. The non-conducted P waves are followed by ventricular paced beats.
